# Bone marrow-derived mesenchymal stem cell-conditioned medium ameliorates diabetic foot ulcers in rats

**DOI:** 10.1016/j.clinsp.2023.100181

**Published:** 2023-03-20

**Authors:** Yi-Feng Xu, Yan-Xiang Wu, Hong-Mei Wang, Cui-Hua Gao, Yang-Yang Xu, Yang Yan

**Affiliations:** a Department of Endocrinology, Air Force Hospital of Northern Theater Command of PLA, China; b Department of Hematology, Air Force Hospital of Northern Theater Command of PLA, China

**Keywords:** Diabetic Foot Ulcers, Conditioned-Medium, Mesenchymal Stem Cells, Therapy

## Abstract

**Objectives:**

This study aimed to explore the effects of bone marrow-derived Mesenchymal Stem Cell-Conditioned Medium (MSC-CM) treating diabetic foot ulcers in rats.

**Methods:**

Models of T2DM rats were induced by a high-fat diet and intraperitoneal injection of STZ in SD rats. Models of Diabetic Foot Ulcers (DFUs) were made by operation on hind limbs in diabetic rats. Rats were divided into four groups (n = 6 for each group), i.e., Normal Control group (NC), Diabetes Control group (DM-C), MSC-CM group and Mesenchymal Stem Cells group (MSCs). MSC-CM group was treated with an injection of conditioned medium derived from preconditioned rats' bone marrow MSCs around ulcers. MSCs group were treated with an injection of rats' bone marrow MSCs. The other two groups were treated with an injection of PBS. After the treatment, wound closure, re-epithelialization (thickness of the stratum granulosums of the skin, by H&E staining), cell proliferation (Ki67, by IHC), angiogenesis (CD31, by IFC), autophagy (LC3B, by IFC and WB; autolysosome, by EM) and pyroptosis (IL-1β, NLRP3, Caspase-1, GSDMD and GSDMD-N, by WB) in ulcers were evaluated.

**Results:**

After the treatment wound area rate, IL-1β by ELISA, and IL-1β, Caspase-1, GSDMD and GSDMD-N by WB of MSC-CM group were less than those of DM group. The thickness of the stratum granulosums of the skin, proliferation index of Ki67, mean optic density of CD31 and LC3B by IFC, and LC3B by WB of MSC-CM group were more than those of DM group. The present analysis demonstrated that the injection of MSC-CM into rats with DFUs enhanced the wound-healing process by accelerating wound closure, promoting cell proliferation and angiogenesis, enhancing cell autophagy, and reducing cell pyroptosis in ulcers.

**Conclusions:**

Studies conducted indicate that MSC-CM administration could be a novel cell-free therapeutic approach to treat DFUs accelerating the wound healing process and avoiding the risk of living cells therapy.

## Highlights

• BMMSC-CM therapy on rats with DFUs enhanced the wound healing process.
• It accelerated wound closure and promoted cell proliferation and angiogenesis.
• It enhancd cell autophagy and reduced cell pyroptosis in ulcers.

## Introduction

In 2021 there are 537 million people living with diabetes. It is predicted that by 2045, 700 million people will suffer from this disease worldwide.^1^ The pooled estimate of the global prevalence of Diabetic Foot Ulcers (DFUs) is approximately 3% in community-based cohorts with a wide variation in rates of major amputation across the world.^2^^,^^3^ DFUs is one of the most severe chronic complications of diabetes with high treatment costs, which can lead to amputation and death. One estimate suggests that between one-third to one-fifth of patients with DM will develop a chronic non-healing wound such as a Diabetic Foot Ucer (DFU) in their lifetime, with an alarming recurrence rate (40% within one year and 65% within five years) and there is no reliable way to predict its occurrence.^4^^,^^5^ The lifetime incidence of foot ulcers in people with diabetes can be as high as 19% to 34%.^6^ Therefore, a large proportion of patients require amputation and expensive treatment, affecting the quality of life of patients. With the DFU market alone estimated to grow from US $7.03 billion in 2019 to US $11.05 billion in 2027, more effective diagnostic and therapeutic strategies must be developed to combat this debilitating disease.^7^^,^^8^

In recent years, stem cell therapy technology has developed rapidly. Mesenchymal Stem Cells (MSCs) have a high self-renewal ability. It's convenient to be collected, and easy to be isolated and cultured for transplantation. MSCs therapy can promote wound healing by reducing inflammation, promoting angiogenesis and granulation tissue formation, and accelerating epithelialization. But some limitation restricts the wide application of MSCs therapy. The main therapeutic mechanism associated with MSCs administration is thought to be the paracrine secretion of a broad spectrum of bioactive factors and extracellular vesicles, commonly referred to as MSC-Conditioned Medium (MSC-CM).^9^

Therefore, the authors performed this study of MSC-CM treating DFUs in rats. The aims of this study were to determine the therapeutic effect of BMMSC-CM treatment on DFUs in rats, and to investigate the possible mechanism of the treatment.

## Methods

### Animal models and groups

Experimental protocols and methods in the current study have been approved by Institutional Animal Care and Use Committee (IACUC) of China Medical University (IACUC Issue n° CMU2022711) and were performed in accordance with the ARRIVE guidelines 2.0.^10^ Male Sprague Dawley (SD) rats weighing 90–100g aged four weeks were obtained from SPF (Beijing) Biotechnology Co.Ltd (SCXK2019-0010). All rats were housed at 25 ± 1°C on a 12h light/dark cycle and fed ad libitum for 1 week before study inception. Animals for diabetes models were then fed with a high-fat diet (40% fat, 40% carbohydrate, and 20% protein) for 8 weeks, and diabetic rat models were generated through a single intraperitoneal injection of streptozotocin (STZ, Sigma, USA) at 30 mg/kg body weight in sodium citrate buffer (pH4.2) after overnight fasting for 15 hours. Blood Glucose (BG) concentration was measured using a drop of tail capillary blood by a glucometer. Fasting Blood Glucose (FBG) ≥ 8.3 mmoL/L after 3 days for 7 days, was indicative of the successful establishment of the T2DM rat model.^11^^,^^12^

DFUs models were created by removing full-thickness skin of 3 × 7 mm from the right hind limb of the diabetic rats, then they were randomly divided into three groups as follows: MSC-CM therapy group (MSC-CM, n = 6), MSCs therapy group (MSCs, n = 6), and diabetes control group (DM-C, n = 6). Normal rats also received an operation for ulcers in the same way and were set as the normal control group (NC, n = 6).

### Cell culture, MSC-CM therapy

Isolation, culture, and identification of MSCs: Bone marrow was collected from both lateral femurs and tibias of one 4-week-old male SD rat weighing 150g. A complete culture medium was prepared, consisting of high glucose Dulbecco's Modified Eagle's Medium (DMEM) with 10% Fetal Bovine Serum (FBS). Cut off the epiphysis at both ends of the femurs and tibias from the joint of the rat with ophthalmic scissors to expose the bone marrow cavity. Flushed the bone marrow out from one end of the bone marrow cavity and then flushed the bone marrow out in the opposite direction from the other end of the bone marrow cavity with culture medium in a 1ml syringe repeatedly, until flushing fluid from the bone marrow cavity became clear. Bone marrow cells were collected by Ficoll-Hypaque density gradient centrifugation. These cells were cultured at 37°C with 5% CO_2_ in a complete culture medium. Nonadherent cells were removed, and a fresh medium was added after 48h of incubation. The medium was changed every 48h or 72h and further propagated the adherent spindle-shaped cells for three passages. BM-MSCs were harvested and identified by flow cytometry as CD73+ CD90+ CD105+ CD34− CD45− HLA-DR− for the expression of MSC markers.^13^

Preparation of MSC-CM: When the confluency of MSCs in 3^rd^ generation reached 80%‒90%, MSCs were cultured in L-DMEM without FBS and penicpstreptomycin for 24h. Then the supernatant was collected, and the dead cells were removed by centrifugation. The medium obtained was concentrated for about 25 times by ultrafiltration, filtered with 0.22 µm microporous membrane filter to remove bacteria. The concentrated conditioned media were frozen and stored in a refrigerator at -80°C until use.

MSC-CM therapy: When the models were created, MSC-CM were injected into four sites around the ulcer of each rat in MSC-CM group, totally 100 µL for each rat. 10^6^ of MSCs were injected into the ulcer of each rat in MSCs group. DM-C group and NC group were injected with the same amount of PBS in the same way.

### Measurement of body weight, wound area and blood glucose level

Digital photographs of wounds were taken on days 0, 3, 7, 10, and 14. Body weight and fasting blood glucose level were determined at day 0, 7 and 14. The wound area was measured using Image-pro Plus 6.0 analysis software (IPP, Media Cybernetics, Inc.) by tracing the wound margin. The wound area rate was calculated as follows: Wound area (%) = ([area of actual wound] / [area of original wound]) × 100.

Histological assessment: At day 14 after therapy, the SD rats were killed and the wound samples (including 2 mm of the surrounding skin of the ulcers) were harvested for histological analysis.

H&E staining: The sections of the wound tissue were stained with Hematoxylin and Eosin (H&E) and the thickness of the stratum granulosums of the skin was measured by Caseviewer Software 2.4 (3DHISTECH Ltd) to detect the hyperblastosis of tissue formation.

ELISA (enzyme-linked immunosorbent assay): The levels of inflammatory factors Interleukin-1β (IL-1β) in ulcers were detected by ELISA kit (Product # abs104566; Absin).

Immunohistochemistry (IHC) and Immunofluorescence Colony (IFC) Staining: The anti-Ki67 antibody (1:300; Product # A16919; ABclonal) IHC, anti-CD31 antibody (1:300; Product # ab182981; ABcam) and the anti-LC3B antibody (1:200; Product # ABS82; Sigma-Aldrich) IFC were performed. Ki67 in ulcers was detected by immunohistochemistry and Proliferation Index (PI) was calculated. PI was calculated as follows: PI = Number of proliferative cells / (Number of proliferative cells + Number of normal cells). CD31 and LC3B were detected by immunofluorescence. IPP software was used to count positively stained cells in immunofluorescence sections, and the Integrated Optical Density (IOD) of positive staining for CD31 or LC3B was calculated and analyzed. Mean Optical Density (MOD) was calculated (MOD = IOD SUM/area) and compared.

Electron microscopy: The ulcer tissue samples were obtained from each group (three samples per group) and cut into small cubes (1 × 1 × 1 mm^3^). Samples were rinsed with Phosphate Buffered Saline (PBS), fixated in 2.5% glutaraldehyde and dehydrated, and sectioned with an ultrathin microtome (Leica, Witzla, Germany), stained with saturated uranyl acetate. Autophagosomes were observed by transmission electron microscope (TEM, H-7650, Hitachi, Osaka, Japan).

Western blot analysis: Total protein was extracted from samples of the wound by Total Protein Extraction Kit (Beyotime Institute of Biotechnology, Shanghai, China) at day 14 posttreatment. Equal amounts of total protein were separated on 10% SDS-PAGE and transferred to nitrocellulose membranes. Membranes were incubated overnight at 4°C with monoclonal antibodies against IL-1β (Product # A1112; Abclonal), LC3B(Product # 14600-1-AP; Proteintech), NLRP3 (Product # A5652; Abclonal), Caspase-1 (Product # A0964; Abclonal), GSDMD (Product # 66387-1-Ig; Proteintech), GSDMD-N (Product # ab215203, Abcam) and GAPDH (Product # 10494-1-AP; Proteintech) (all 1:1000). Then, the membranes were incubated with HRP-conjugated anti-rabbit (1:5000; Product # S0001; Affinity).

### Statistical analysis

Data are shown as means ± Standard Deviation (SD). Before analysis, the data were tested for normality of distribution using the Kolmogorov-Smirnov test. For normally distributed data, differences between groups were analyzed using the Least-Significant Difference test (LSD) and repeated measurement analysis. A value of p < 0.05 was considered significant. SPSS 22.0 (IBM) was used for statistical analyses.

## Results

### Identification of BM-MSCs characteristics

**Fig. 1:**
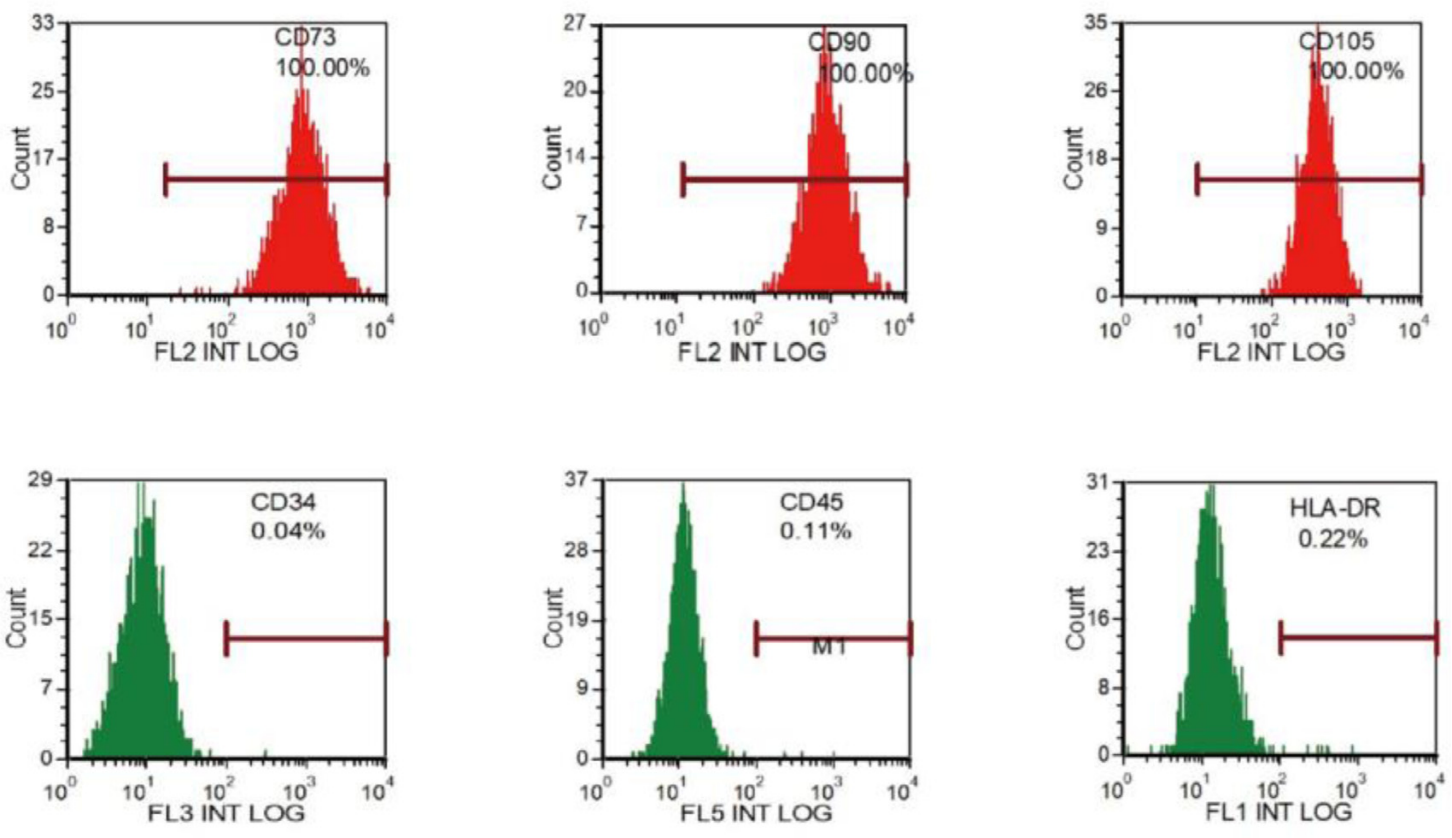
Characterization of rat BM-MSCs. Cell surface markers of MSCs were assessed using flow cytometry. MSCs expressed CD73, CD90 and CD105, but not CD34, CD45 or HLA-DR.

Isolated cells were plastic-adherent in culture and displayed a typical fibroblast morphology. Flow cytometry analysis showed that the BM-MSCs slightly expressed hematopoietic CD markers CD34 (0.04%), CD45 (0.11%) and HLA-DR (0.22%), and completely expressed mesenchymal CD markers CD73, CD90, and CD105 (100%), indicating that the cultured cells possessed the MSCs characteristics (Fig. 1).

### Measurement of body weight, wound area, and blood glucose levels

The body weight of DFUs was higher than that of NC group. There were no differences in body weight among DM-C, MSC-CM, and MSCs groups (Table 1)

**Table 1:** Body weight of the rats before and after therapy.

Group	0 d (g)	7 d (g)	14 d (g)
NC	352±29^a^	381±36^a^	391±42^a^
DM-C	401±25	407±27	416±24
MSC-CM	403±19	411±22	420±23
MSCs	398±26	406±23	414±18

a p < 0.05 compared with the other three groups.

**Fig. 2:**
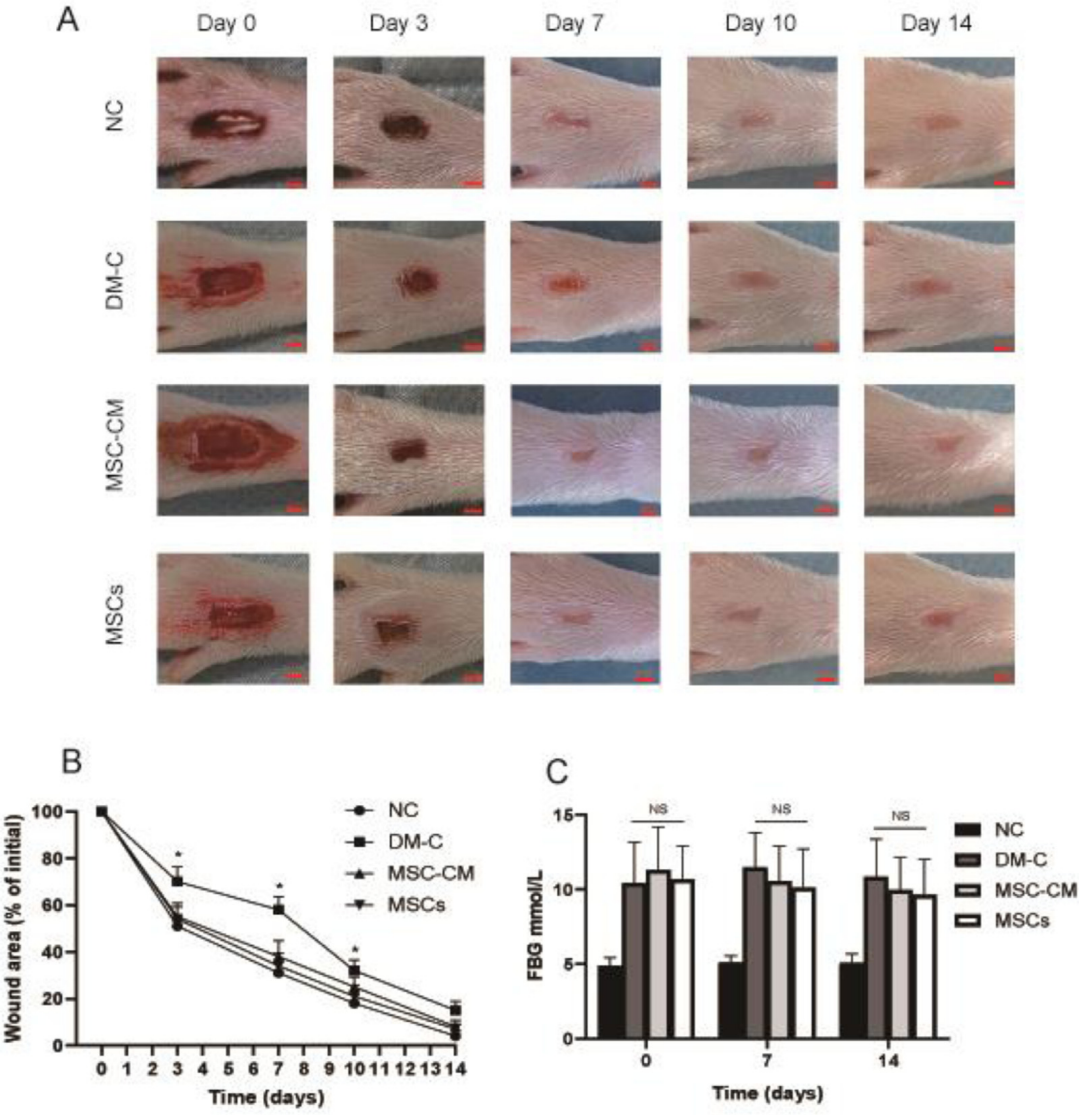
Wound area and fasting blood glucose levels. (A) Effect on DFUs in rats after treatment (2 mm). (B) Wound area rate. At day 3, 7 and 10, wound area rate of DM-C group was higher than those of the other three groups. (*p < 0.05). (C) Blood glucose levels. There were no significant differences in fasting blood glucose levels among DM-C, MSC-CM and MSCs group.

Both MSC-CM and MSCs therapy enhanced wound healing. Wounds of MSC-CM and MSCs groups exhibited accelerated wound closure compared with wounds of DM-C group on day 3, day 7 and day 10 (p < 0.05). There were no significant differences in the wound area between MSC-CM and MSCs groups (Fig. 2 A‒B; Table 2).

**Table 2:** Wound area of the rats before and after therapy.

Group	0 d (%)	3 d (%)	7 d (%)	10 d (%)	14 d (%)
NC	100	51±4	31±5	18±4	4±1
DM-C	100	70±6^a^	58±6^a^	32±5^a^	15±4^a^
MSC-CM	100	55±6	38±7	22±4	8±3
MSCs	100	54±5	34±6	21±5	7±2

a p < 0.05 compared with the other three groups.

Fasting blood glucose levels of DM-C, MSC-CM, and MSCs group was higher than that of NC group. There were no significant differences in blood glucose levels among DM-C, MSC-CM, and MSCs groups (Fig. 2C; Table 3).

**Table 3:** FBG of the rats before and after therapy.

Group	0 d (mmoL/L)	7 d (mmoL/L)	14 d (mmoL/L)
NC	4.88±0.55^a^	5.12±0.46^a^	5.07±0.62^a^
DM-C	10.43±2.75	11.47±2.33	10.84±2.54
MSC-CM	11.32±2.88	10.57±2.34	9.97±2.18
MSCs	10.68±2.23	10.12±2.59	9.65±2.37

a p < 0.05 compared with the other three groups.

### Histological assessment

**Fig. 3:**
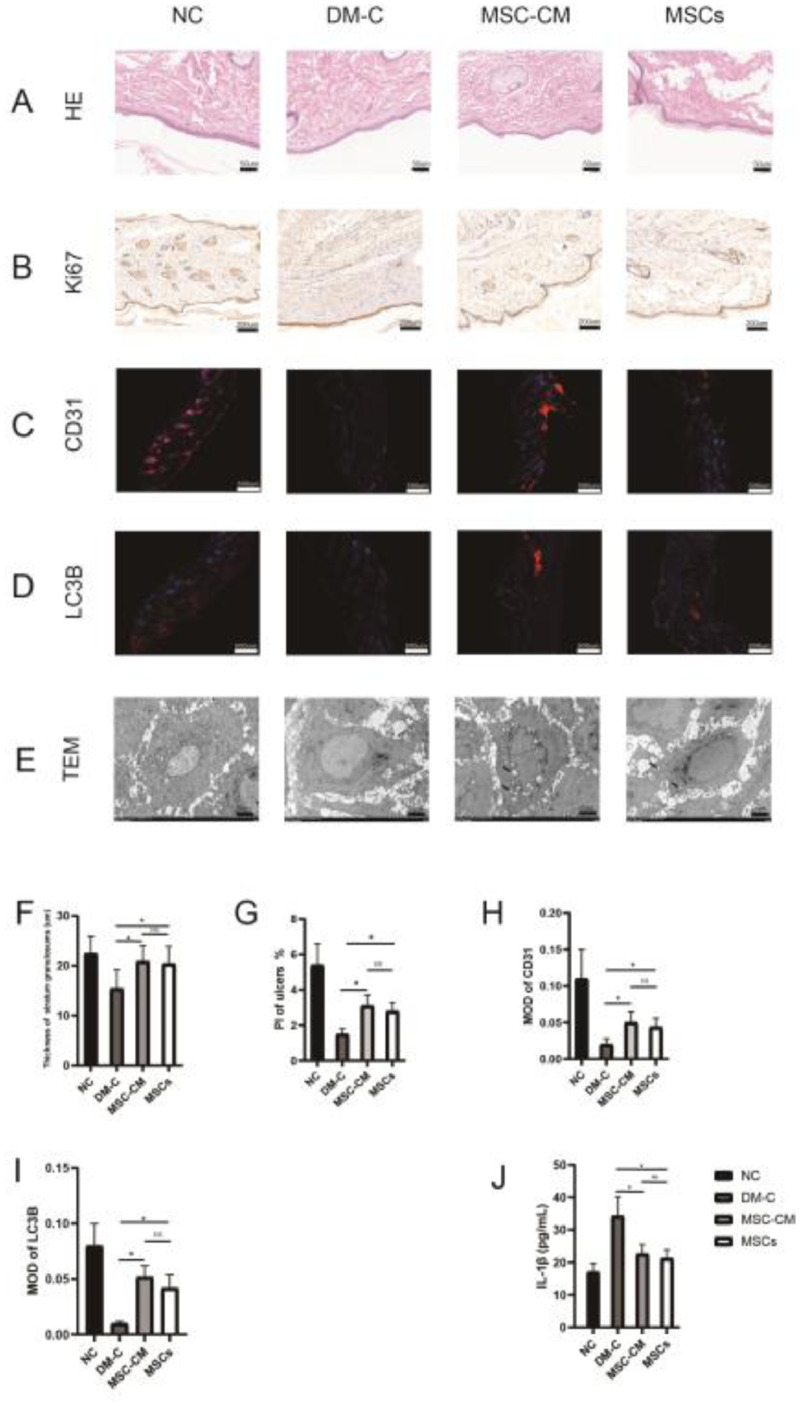
Histological assessment of the skin of ulcer specimens from rats at day 14 after therapy. (A) H&E-stained sections (50 µm). (B) IHC of Ki67 in the skin of ulcer specimens (50 µm). (C) IFC of CD31 in the skin of ulcer specimens (500 µm). (D) IFC of LC3B in the skin of ulcer specimens (500 µm). (E) TEM of the skin of ulcer specimens (2 µm). Autophagosomes (arrow) could be seen in MSC-CM and MSCs group but could hardly be found in DM-C group. (F) The thickness of the stratum granulosums of the skin. The thickness of the stratum granulosums of the skin in MSC-CM or MSCs group was thicker than that in DM-C group (*p < 0.05). (G) PI from ki67 in ulcers. PI of MSC-CM or MSCs group was more than that of DM group (*p < 0.05). (H) MOD from CD31 in ulcers. MOD from CD31 of MSC-CM or MSCs group was higher than that of DM group (*p < 0.05). (I) MOD from LC3B in ulcers. MOD from LC3B of MSC-CM or MSCs group was higher than that of DM group (*p < 0.05). (J) IL-1β levels in ulcers. IL-1β level in ulcers of MSC-CM or MSCs group was lower than that of DM-C group (*p < 0.05).

H&E staining: The thickness of the stratum granulosums of the skin in MSC-CM or MSCs group was thicker than that in DM-C group (p < 0.05). There were no significant differences in the thickness of the stratum granulosums between MSC-CM and MSCs groups (Fig. 3 A‒F, Table 4).

**Table 4:** Histology parameters of wound at day 14.

Group	Thickness of stratum granulosums (µm)	PI (%)	CD31	LC3B	IL-1β (pg/mL)
NC	22.5±3.4	5.4±1.2	0.11±0.04	0.08±0.02	17.1±2.5
DM-C	15.4±3.8^a^	1.5±0.3^a^	0.02±0.01^a^	0.01±0.002^a^	34.3±5.8^a^
MSC-CM	20.9±3.2	3.1±0.6	0.05±0.02	0.05±0.01	22.7±2.8
MSCs	20.4±3.5	2.8±0.5	0.04±0.01	0.04±0.01	21.3±2.5

a p < 0.05 compared with the other three groups.

ELISA: IL-1β level in ulcers of MSC-CM or MSCs group was lower than that of DM-C group (p < 0.05). There were no significant differences in IL-1β levels of ulcers between MSC-CM and MSCs groups (Fig. 3J, Table 4).

IHC and IFC: PI from ki67 in ulcers of MSC-CM or MSCs group was more than that of DM group (p < 0.05). There were no significant differences with PI in ulcers between MSC-CM and MSCs groups (Fig. 3 B and G). MOD from CD31 in ulcers of MSC-CM or MSCs group was higher than that of DM group (p < 0.05). There were no significant differences with CD31 in ulcers between MSC-CM and MSCs groups (Fig. 3 C and H). MOD from LC3B in ulcers of MSC-CM or MSCs group was higher than that of DM group (p < 0.05). There were no significant differences with LC3B in ulcers between MSC-CM and MSCs groups (Fig. 3 D and I, Table 4).

Electron microscopy: Treatment with MSC-CM or MSCs induced the appearance of autophagosomes in the cells. Autophagosomes could hardly be found in DM-C group (Fig. 3E).

**Fig. 4:**
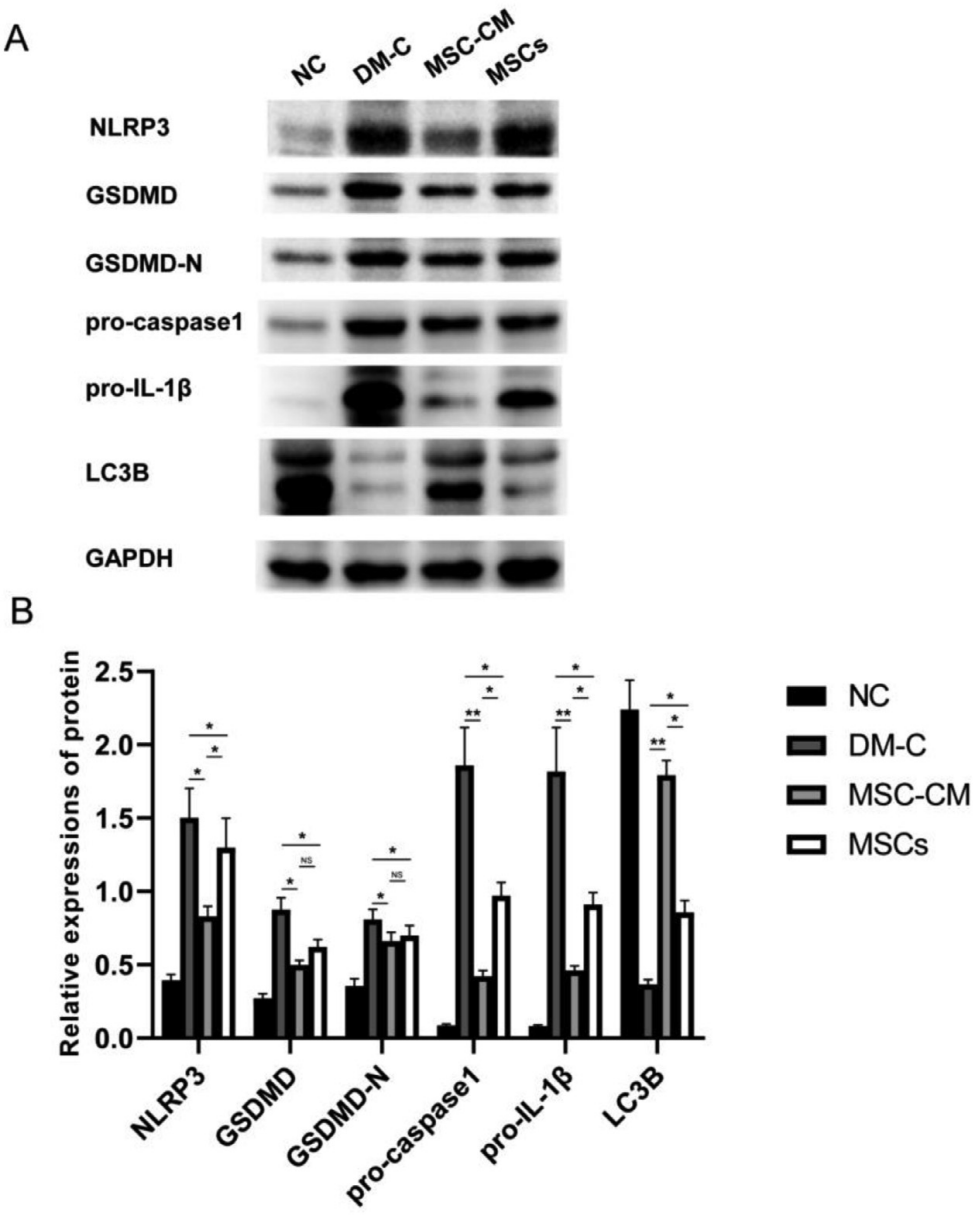
Western blot analysis. (A) Western blot analysis of NLRP3, GSDMD, GSDMD-N, proCaspase-1, pro-IL-1β, LC3B and GAPDH expression in wound site at day 14 of four groups. (B) The quantification of relative expressions of protein of NLRP3, GSDMD, GSDMD-N, proCaspase-1, pro-IL-1β and LC3B by Western blot. The expressions of NLRP3, GSDMD, GSDMD-N, proCaspase-1 and pro-IL-1β in MSC-CM or MSCs group decreased obviously compared with those in DM-C group. The expressions of LC3B in MSC-CM group were higher than those in MSCs or DM-C group. (*p < 0.05, **p < 0.01).

Western blot analysis: The relative expressions of protein of NLRP3, GSDMD, GSDMD-N, proCaspase-1 and pro-IL-1β in MSC-CM or MSCs group decreased obviously compared with those in DM-C group. The expressions of NLRP3, proCaspase-1 and pro-IL-1β in MSC-CM group were less than those in MSCs group. The relative expressions of protein of LC3B in MSC-CM group were higher than those in MSCs or DM-C group (Fig. 4).

## Discussion

Stem cell therapy for the treatment of DFUs has been a topic of much interest recently. Murine models of diabetes have found that stem cells derived from umbilical, adipose, smooth muscle, and bone marrow or in combination therapies with MSCs accelerated wound healing.^14^, ^15^, ^16^, ^17^, ^18^ BM-MSCs transplantation is a therapeutic way for DFUs, and intramuscular transplantation has been proven to have the probably best efficacy.^19^ However, currently, there are some limitations that hinder the widespread use of MSCs, such as spontaneous changes in properties and behavior, formation of malignant tumors, transmission of infectious diseases^20^^,^^21^ and so on.

Recent studies have shown that engrafted MSCs do not survive for the long term, suggesting that the benefits of MSC therapy might be attributable to their secreted factors. The function of mesenchymal stem cells to secrete protective factors was first discovered by Gnecchi et al.^22^ At present, many studies have confirmed that the paracrine effect is the main mechanism of MSCs therapy.^23^, ^24^, ^25^, ^26^ CM represents a fully regenerated milieu and the vesicular component of the cell-derived secretome. A growing body of literature recently has drawn attention to the plethora of bioactive factors produced by MSCs, including growth factors, cytokines, microRNAs, exosomes, and proteasomes, which may play important roles in the regulation of many physiological processes. The use of CM may have considerable potential advantages over living cells in terms of manufacturing, handling, storage, product shelf life, and their potential as ready-to-use biotherapeutics.^27^^,^^28^ It has been demonstrated that MSC-CM is sufficient to improve multiple pathophysiological biomarkers significantly and to be effective in the transplantation of the corresponding MSCs in many different animal models. BMMSC-CM has been used to treat many diseases such as spinal cord injury, cerebrovascular disease, lung injury, and so on.^29^, ^30^, ^31^

Pyroptosis is the process of inflammatory cell death. There are two major pathways for pyroptosis: canonical and noncanonical pyroptosis. In the canonical pyroptosis pathway, activated Caspase-1 cleans GSDMD protein, and the cleaved GSDMD produces an independent domain fragment as the N-terminal. GSDMD-N binds to the cell membrane, forms pores, and the cytoplasmic membrane is destroyed, resulting in pyroptosis and inducing inflammatory cell death.^32^ At the same time, activated caspase-1 cleaves the precursor of IL-1β to form active IL-1β, which is released to the outside of the cell through the pores and causes an inflammatory response. In vivo autophagy is a protective response that inhibits intracellular signaling and regulates the activation of inflammasomes by removing dysfunctional mitochondria.^33^ Impaired autophagy can activate NLRP3 inflammasome to trigger canonical pyroptosis^34^^,^^35^ and expand the inflammatory effect. Studies have suggested that pyroptosis is associated with the onset of diabetes and its complications.^36^^,^^37^ So reducing pyroptosis may have therapeutic effects on diabetic complications.

Some studies conducted about MSC-CM treating skin wounds. One study showed that the concentrated hypoxia-preconditioned adipose mesenchymal stem cell-conditioned medium could accelerate the skin wound healing in a rat full-thickness skin defect model, however, this study did not involve the mechanism of the treatment.^38^ A study in vitro showed that BMMSC-CM of rats could improve the proliferation and migration of keratinocytes in a diabetes-like microenvironment by decreasing High Glucose (HG) and/or Lipopolysaccharide (LPS) induced Reactive Oxygen Species (ROS) overproduction and reversing the downregulation of phosphorylation of MEK 1/2 and Erk 1/2.^39^ A recent study showed that adipose-derived stem cell CM could accelerate wound healing and hair growth in SD rats with burn wounds on the dorsal, but this study also did not reveal the mechanism of the treatment.^40^ In the present study, HE and Ki67 staining suggested that the treatment of MSC-CM promoted the proliferation of skin tissue, CD31 staining suggested that the treatment promoted the proliferation of blood vessels and increased the local blood supply. Electron microscopy showed that the treatment promoted cell autophagy. Autophagy was enhanced by promoting the expression of LC3B. The inflammatory state was improved by reducing the levels of NLRP3 and IL-1β. Caspase-1 was inhibited, and the expression of GSDMD-N was reduced, thereby cell pyroptosis was inhibited. The curative efficacy of MSC-CM therapy was similar to that of MSCs.

MSC-CM therapy, namely the use of cell-free therapy, has considerable advantages over cell-based applications. MSC-CM therapy resolves several safety concerns that may be associated with living cell transplantation including tumorigenicity, embolism, immune compatibility, and spread of infections. MSC-CM can be stored for long periods of time without losing much product potency.^41^^,^^42^ MSC-CM therapy does not require invasive cell collection procedures, and it is more economical, practical, and suitable for clinical applications.^43^ MSC-CM can be used in specific laboratory conditions, and produced in large quantities to provide controlled bioactive factors.

Factors secreted by different MSCs may be different, such as Adipose-Derived Stem Cells-CM (ADSC-CM) expresses Vascular Endothelial Growth Factor (VEGF), Nerve Growth Factor (NGF), Stem Cell Factor (SCF), and Hepatocyte Growth Factor (HGF), while human Umbilical Cord Perivascular Cell-CM (hUCPVC-CM) expressed no SCF or HGF.^44^^,^^45^ There were also differences between the composition of ADSC-CM and BMMSC-CM.^46^ In the present study, the authorsdid not detect the components of the MSC-CM. In order to standardize the production of CM from each MSC type, further studies on culture conditions, culture duration, culture medium, and supplements, and the criteria for the composition of MSC-CM are required.

## Conclusion

BMMSC-CM is effective in the treatment of DFUs in type 2 diabetic rats. BMMSC-CM can promote the healing of DFUs by inhibiting inflammation, enhancing autophagy, and reducing pyroptosis. These findings highlight a potential therapeutic method of BMMSC-CM for the treatment of DFUs, avoiding the risk of living cell therapy.

## Authors' contributions

All authors contributed to the conception of the work. Yi-Feng Xu contributed to study design, experiment performing, data analysis and wrote the manuscript. Yan-Xiang Wu and Hong-Mei Wang contributed to review & editing. Cui-Hua Gao, Yang-Yang Xu, and Yang Yan contributed to experiment performing and the data acquisition. Yi-Feng Xu and Yan-Xiang Wu contributed equally to this work. All of the authors have given final approval and agree to be responsible for all aspects of the work, ensuring accuracy and precision.

## Conflicts of interest

The authors declare no conflicts of interest.
